# Hemodynamic Monitoring in the Critically Ill Patient – Current Status and Perspective

**DOI:** 10.3389/fmed.2015.00044

**Published:** 2015-08-03

**Authors:** Samir G. Sakka

**Affiliations:** ^1^Department of Anesthesiology and Operative Intensive Care Medicine, Medical Center Cologne Merheim, University Witten/Herdecke, Cologne, Germany

**Keywords:** hemodynamics, monitoring, intraoperative, cardiac output, fluid therapy, catecholamines

## Abstract

In the critically ill patient, early and effective hemodynamic management including fluid therapy and administration of vasoactive drugs to maintain vital organ perfusion and oxygen delivery is mandatory. Understanding the different approaches in the management of critically ill patients during the resuscitation and further management is essential to initiate adequate context- and time-specific interventions. Treatment of hemodynamic variables to achieve a balance between organ oxygen delivery and consumption is the cornerstone. In general, cardiac output is considered a major determinant of oxygen supply and thus its monitoring is regarded helpful. However, indicators of oxygen requirements are equally necessary to assess adequacy of oxygen supply. Currently, more and more less or even totally non-invasive monitoring systems have been developed and clinically introduced, but require validation in this particular patient population. Cardiac output monitors and surrogates of organ oxygenation only enable to adequately guide management, as patient’s outcome is determined by acquisition and interpretation of accurate data, and finally suitable management decisions. This mini-review presents the currently available techniques in the field of hemodynamic monitoring in critically ill patients and briefly summarizes their advantages and limitations.

Since its introduction in the early 1970s the pulmonary artery catheter (PAC) was widely used and regarded as the “holy grail” in hemodynamic monitoring (1). This device, for the first time, allowed to measure extended hemodynamic variables, particularly cardiac output, cardiac filling pressures, and global oxygen transport, in the critically ill patient at the bedside. However, in 1996 when Connors et al. (2) reported in an observational study based on a matched-pair analysis that the use of a PAC was associated with an increased mortality and increased utilization of resources, even a moratorium for the PAC was called for by some authors. Over the following years, several large randomized controlled trials could not confirm these findings, however, they failed to demonstrate improved outcome in patients monitored with the PAC (3–6). Thus, up to date no damage but also no benefit on outcome by using a PAC has been documented. Consequently, a decline in PAC use over the following years developed (7, 8). Although potentially life-threatening complications during the catheter placement (e.g., cardiac arrhythmias and mechanical complications) are well described, data showed that negative impact on outcome is based on the period after the initial phase suggesting that correct measurement of variables and decisions made are more critical. Nevertheless, the PAC may still provide important information especially in patients with pulmonary arterial hypertension and right ventricular failure, however, there is consensus that the PAC should not be routinely used as the primary means of advanced monitoring (9).

In general, no specific monitoring device has been found to reduce mortality in critically ill patients (10), which is not surprising as the conclusions drawn from the hemodynamic variables obtained and the treatment induced are finally determining. This statement is confirmed insofar, as there is a convincing body of evidence that goal-directed optimization of advanced hemodynamic variables reflecting global blood flow improves patients’ outcome (11, 12).

During the time, when the PAC came under intense debate, more and more alternatives and especially less-invasive techniques were developed and evaluated in experimental and clinical studies. Besides transpulmonary indicator (e.g., thermal, lithium), dilution techniques, less-invasive technologies like esophageal Doppler and uncalibrated pulse contour analysis (based on a peripheral arterial signal) were introduced. Furthermore, as a logical step, several manufacturers have promoted and introduced totally non-invasive devices for advanced hemodynamic monitoring: e.g., thoracic bioimpedance, bioreactance, and ultrasound techniques. Although described first in the early 1990s, non-invasive systems for assessment of radial artery pressure curves based on the volume clamp method according to the Peñaz principle and applanation tonometry are currently intensively studied in critically ill patients.

In general, cardiac output as major and flow determinant variable of systemic oxygen delivery is often considered helpful in the management of critically ill patients. However, measurement of cardiac output *per se* is only of limited value as its interpretation (Frank-Starling relation) requires simultaneous information on cardiac preload and/or fluid responsiveness (as an *a priori* estimate of the impact of fluid loading on cardiac output). However, some but not all of the techniques listed below simultaneously provide information on cardiac output and cardiac preload or fluid responsiveness, respectively. Since not intended here and thus without claiming completeness, Table 1 provides an updated overview on the currently available systems (13).

**Table 1:** **Overview on various hemodynamic monitoring devices**.

Modality	Device	Equipment	Limitations
Right heart catheterization	Pulmonary artery catheter	Thermistor-tipped balloon catheter	Invasiveness
		Special types enabling continuous monitoring of cardiac output and/or mixed venous saturation, right heart ejection or pacing modalities	Mechanical complications, arrhythmias, infections

Central venous oxygen saturation	Central venous catheter	Intermittent or continuous measurement of O_2_-saturation	Mechanical complications, arrhythmias, infections
			Not equal to mixed venous O_2_-saturation

Pulse pressure
Calibrated	PiCCO^R^	Thermistor-tipped arterial catheter	Hematoma, vessel occlusion with ischemia,
	EV100/VolumeView^R^	Thermistor-tipped arterial catheter	embolism, infections
	LiDCO^R^	Arterial catheter and lithium sensor	see above (cave: lithium levels)
Uncalibrated	FloTrac/Vigileo^R^	Specific arterial kit	Hematoma, vessel occlusion with ischemia,
	LiDCO Rapid^R^	Specific arterial kit	embolism, infections
	PulsioFlex^R^	Arterial catheter	
	PRAM (MostCare^R^)	Specific arterial kit	
Non-invasive	Nexfin^R^	Finger pressure cuff	Local swelling, peripheral ischemia
	CNAP^R^	Finger pressure cuff, oscillometry	Limited accuracy of measurement of absolute cardiac output
	T-Line^R^	Radial applanation tonometry	

Ultrasound			
Doppler			
Esophageal Doppler	CardioQ^R^	Esophageal probe	Limited accuracy of measurement of absolute cardiac output
Transthoracic Doppler	USCOM^R^	Transthoracic probe	
Echocardiography	ClariTEE^R^	Disposable monoplane echo probe	Limited duration of placement, findings operator and experience dependent

Fick principle			
Partial CO_2_ rebreathing	NICO^R^	Rebreathing loop	No information on cardiac preload
Dye dilution	DDG analyzer^R^	Cutaneous sensor	Absolute measurement of cardiac output limited

Bioimpedance/-reactance			
Thoracic bioimpedance	BioZ^R^	Specific electrodes	Limited accuracy of measurement of absolute cardiac output
Thoracic bioreactance	NICOM^R^	Specific electrodes	
Electrical velocimetry	Aesculon^R^	Specific electrodes	

Plethysmography			
Plethysmogram variability	MASIMO^R^	Specific transcutaneous probe	Difficulties in data acquisition in critically ill patients
			Accuracy for measurement of absolute cardiac output limited

Before providing an overview on these technologies, briefly discussing their strengths and weaknesses, an update on the *status quo* should be given. Today, as reported in a current survey from Switzerland, 56% of intensivists use transpulmonary thermodilution (1% lithium dilution), 31% a PAC, 9% echocardiography, and 3% an uncalibrated pulse contour system, respectively (14). Notably, pulse pressure variation (PPV) and stroke volume variation (SVV) are mostly used in the Australia and New Zealand, while esophageal Doppler technology is preferred by anesthetists in the UK for guiding intraoperative fluid management (15).

In order to assess adequacy of oxygen delivery, central venous oxygen saturation (ScvO_2_) as a measure of oxygen transport has been proposed since the trial by Rivers et al. in 2001 (16). However, the positive results of a ScvO_2_-guided treatment on outcome in patients with sepsis as reported in this monocentric trial were recently challenged (17). From physiology, central and mixed venous oxygen saturation cannot be equal, however, the first as obtained in the V. cava superior can be obtained less invasively and is commonly used as a surrogate for the match/mismatch between oxygen supply and demand. As some authors suggest an average difference of 7 ± 4% exists between both variables (18), there is larger difference especially in low cardiac output states (19). Consequently, trending of ScvO_2_ is probably more clinically relevant (18) and high values (≥77.4%) should alert in terms of systemic inflammation, lactic acidosis, and increase in mortality (20).

Still, thermodilution techniques are regarded as clinical gold standard for the measurement of cardiac output in critically ill patients. Transpulmonary thermodilution has been shown to be equivalent in accuracy when compared to pulmonary artery thermodilution (21) which is often considered as clinical gold standard. Volumetric parameters (i.e., intrathoracic blood volume, global end-diastolic blood volume), which are assessed by the transpulmonary technique, are regarded superior to static parameters of cardiac preload, e.g., cardiac filling pressures (22). Treatment algorithms using transpulmonary indicator dilution have been described to be associated with a reduction in ICU stay, less organ dysfunction, and earlier ready for discharge from the ICU (21). Critically ill patients with subarachnoid hemorrhage, patients after major burn trauma or cardiac arrest were found to have more stable organ function when managed by transpulmonary thermodilution with integrated pulse contour analysis (21). So far, large randomized, controlled studies in the ICU using this system are missing. More recently, flow-directed protocols for intraoperative hemodynamic management using dynamic parameters of fluid responsiveness, i.e., PPV and SVV, have been found to significantly reduce perioperative complication rates, especially in patients undergoing major abdominal surgery (23, 24). To date, PPV is the most reliable predictor of fluid responsiveness in critically ill patients undergoing mechanical positive-pressure ventilation without cardiac arrhythmias (25). However, predictive properties of PPV with respect to fluid responsiveness may be even higher when considering amplitude of airway pressures (26). Future studies are warranted to show in how far integration of respiratory variables (possibly in an automated manner) will improve the predictive properties of PPV. Although in a lower extent, PPV may even be helpful for estimating fluid responsiveness in spontaneously breathing patients as long as no forced respiratory maneuvers occur (27).

Over the last few years, more systems based on uncalibrated pulse contour analysis for a beat-to-beat monitoring of cardiac output are emerging. On the first glance attractive due to the fact that peripheral (e.g., radial) arterial cannulation and without need for a reference method, data are conflicting and reliability of measuring absolute cardiac output correctly is still a matter of clinical studies (28–30). However, trending of cardiac output by an uncalibrated technique has been reported to be more reliable (31). Although intraoperative use of such systems has been found to reduce complication rates, further studies are mandatory to assess their accuracy (32) and potential benefit by implementing in the ICU.

One clinically increasingly attractive technique is the passive leg raising (PLR) maneuver while using a continuous monitoring system which allows real-time following of its effects (33). PLR is a test that predicts whether cardiac output will increase with volume expansion, i.e., around 300 mL of blood from the lower body to the heart. Since no external fluid is given, hemodynamic effects are rapidly reversible and there is no risk of fluid overload. However, several rules should be followed. First, PLR should start from the semi-recumbent and not the supine position as blood from the splanchnic region is mobilized by adding trunk lowering. Second, the PLR effects must be assessed by a direct measurement of cardiac output and not by the simple measurement of blood pressure. Furthermore, the cardiac output monitoring technique during PLR must be able to detect short-term and transient changes since the PLR effects may vanish quickly. For instance, arterial pulse contour analysis, echocardiography, esophageal Doppler, or contour analysis of the volume clamp-derived arterial pressure can be used. Fourth, cardiac output must be measured not only before and during PLR but also after PLR when the patient is back in the semi-recumbent position. As, pain, cough, discomfort, and awakening could provoke adrenergic stimulation, resulting in mistaken interpretation of cardiac output changes, PLR must be performed by adjusting the bed and not by manually raising the patient’s legs.

One step further is the use of totally non-invasive techniques for continuous measurement of blood pressure and cardiac output in critically ill patients. Finger-cuffed systems (34–37) which by pulse contour analysis allow continuous monitoring of cardiac output or radial artery applanation for blood pressure measurement (38, 39) are currently evaluated in the ICU. Noteworthy, some of these systems use a reference (e.g., oscillometry). Although promising data could be obtained over the last years, their routine use in severely ill patients cannot be advocated at this moment.

As a classical Doppler principle-based technology, assessment of cardiac output by esophageal flow probe requires the velocity-time-integral and the vessel cross-sectional area (40). Thus by definition, the esophageal Doppler technique can merely obtain flow in the descending aorta and measurement of absolute cardiac output is not possible. Furthermore, assumption about the aortic size may be erroneous. Moreover, the system uses the peak velocity a surrogate of cardiac contractility and the corrected flow time, i.e., a dynamic estimate of preload. While earlier studies reported positive influence on length of stay or complications in patients undergoing colorectal surgery, newer trials did not show any benefit from such monitoring (41). Data in critically ill patients are rare and warrant further evaluation (42). Noteworthy, movements may affect the precision of readings derived from the esophageal Doppler. Another approach is a supra-sternal ultrasound probe using the flow profile of the ascending aorta. As mentioned above, two main sources may explain the inferiority to thermodilution techniques for measurement of cardiac output which are related to the Doppler measurement of aortic blood flow (i.e., the velocity time interval) and estimation of the aortic cross-sectional area which is derived from an algorithm based on patient size and has nothing to do with Doppler. Data suggest that this technique has the ability to track cardiac output over time properly, however, factors like patient age has significant impact on its accuracy (43).

Although transesophageal echocardiography is not a classical monitoring technique and normally applicable intermittently, a miniaturized (5.5 mm diameter), disposable transesophageal echocardiography probe which may be kept *in situ* for up to 72 h has recently been introduced into the market. Although this method is gaining popularity as a continuous mode of echocardiographic assessment in critical care, it is clearly operator-dependent and needs further evaluation in anesthesia and intensive care (44).

A complete other approach are non-invasive Fick-principle-based techniques. For instance, transcutaneous assessment of soluble indicators (e.g., indocyanine green) has been suggested for measurement of cardiac output. In this technique, (peripheral) venous indicator injection and downstream concentration measurement (i.e., by a finger sensor) allow recording concentration-time curves after adequate mathematical analysis cardiac output. However, the indicator concentration is obtained indirectly from optical densities and the relation between both has primarily been derived in healthy volunteers. Since absolute concentrations are mandatory for correct assessment of cardiac output and since physical properties of the skin and other tissues may significantly differ from healthy humans, data in critically ill patients were not convincing (45). Another Fick-principle-derived technique is the carbon dioxide rebreathing system. Introduced in 1999 (46), the system uses the differential Fick partial rebreathing technique to measure cardiac output in intubated and mechanically ventilated patients. Decreased correlation between this technique and pulmonary artery thermodilution has been reported in high cardiac output states, low minute ventilation, increased intrapulmonary shunt, and severe chest trauma (47). Besides these drawbacks, the system does not provide information on cardiac preload and, thus, its use particularly in critically ill patients cannot be recommended.

Both, bioreactance and bioimpedance are non-invasive methods for estimation of cardiac output. Known for many decades, thoracic bioimpedance allows continuous estimation of cardiac output based on the specific electric resistance of the thorax which changes during the cardiac cycle. A small current is transmitted between electrodes placed on the lower thorax and neck, and the voltage is measured. The electric impedance is derived from the voltage, and is inversely proportional to aortic blood flow. The accuracy of thoracic bioimpedance is affected by alterations in extravascular lung water, respiratory cycle-dependent alterations in venous blood flow, increased aortic stiffness, interference from the mechanical ventilator, and movement of the electrodes. Data on the validity of thoracic bioimpedance in relation to thermodilution estimation of cardiac output have been conflicting and refinements in this technique are required (48). A similar approach, electrical velocimetry (49, 50), is a bioimpedance method of CO determination measuring changes in transthoracic impedance during cardiac ejection to calculate stroke volume. The device is based on emission of a high-frequency (50 kHz) and low-amperage (2 mA) alternating electrical current of constant amplitude via a pair of surface electrodes across the left side of the thorax. So far, inconsistent data in critically ill patients were reported (51, 52).

While the bioimpedance measures changes in amplitude, bioreactance relies on changes in signal frequency. Thoracic biorectance is more refined than thoracic bioimpedance as the ability to accurately measure phase shifts is less dependent on external noise and electrical interference. First data comparing thoracic biorectance to thermodilution techniques suggests that the methods are comparable (53, 54). However, adequate clinical studies are still required to validate the utility of bioreactance and bioimpedance in critically ill patients.

Last but not least, pulse oximetry as a fully non-invasive technique may be a helpful tool in assessing hemodynamics (55). In principle, plethysmographic waveform analysis requires specific tools and software that are not yet widely available. The plethysmographic variability index is based on perfusion index variations during the respiratory cycle and allows automated and continuous calculation of the respiratory variations in the pulse oxymeter waveform amplitude. Data from the operative setting were promising, however, the system is less reliable in critically ill patients and in about one-tenth of vasopressor-dependent patients no adequate signal quality could be obtained (56).

In conclusion, independently the methodological considerations and requirements to guarantee adequate accuracy, calibrated systems are still most reliable for the measurement of cardiac output in critically ill patients. However, the monitoring system *per se* is not outcome relevant but the underlying treatment algorithms are more important. Up to date, it seems indicated that with higher patients’ morbidity application of non-invasive techniques should be held restrictive (57). While without any doubt existing monitoring devices will be refined and new techniques developed, the future will show which treatment strategy does match the best requirements to improve outcome of critically ill patients.

## Conflict of Interest Statement

Samir G. Sakka is a member of the Medical Advisory Board of PULSION Medical Systems SE, Feldkirchen, Germany.
